# Screening the Drug:H^+^ Antiporter Family for a Role in Biofilm Formation in *Candida glabrata*

**DOI:** 10.3389/fcimb.2020.00029

**Published:** 2020-02-04

**Authors:** Rui Santos, Mafalda Cavalheiro, Catarina Costa, Azusa Takahashi-Nakaguchi, Michiyo Okamoto, Hiroji Chibana, Miguel C. Teixeira

**Affiliations:** ^1^Department of Bioengineering, Instituto Superior Técnico, Universidade de Lisboa, Lisbon, Portugal; ^2^Biological Sciences Research Group, Institute for Bioengineering and Biosciences, Instituto Superior Técnico, Lisbon, Portugal; ^3^Medical Mycology Research Center, Chiba University, Chiba, Japan

**Keywords:** *Candida glabrata*, drug:H^+^ antiporters, biofilm formation, CgTpo4, CgDtr1

## Abstract

Biofilm formation and drug resistance are two key pathogenesis traits exhibited by *Candida glabrata* as a human pathogen. Interestingly, specific pathways appear to be in the crossroad between the two phenomena, making them promising targets for drug development. In this study, the 10 multidrug resistance transporters of the Drug:H^+^ Antiporter family of *C. glabrata* were screened for a role in biofilm formation. Besides previously identified players in this process, namely CgTpo1_2 and CgQdr2, two others are shown to contribute to biofilm formation: CgDtr1 and CgTpo4. The deletion of each of these genes was found to lead to lower biofilm formation, in both SDB and RPMI media, while their expression was found to increase during biofilm development and to be controlled by the transcription factor CgTec1, a predicted key regulator of biofilm formation. Additionally, the deletion of *CgDTR1, CgTPO4*, or even *CgQDR2* was found to increase plasma membrane potential and lead to decreased expression of adhesin encoding genes, particularly *CgALS1* and *CgEPA1*, during biofilm formation. Although the exact role of these drug transporters in biofilm formation remains elusive, our current model suggests that their control over membrane potential by the transport of charged molecules, may affect the perception of nutrient availability, which in turn may delay the triggering of adhesion and biofilm formation.

## Introduction

The human opportunistic pathogen, *Candida glabrata*, is responsible for an estimated death rate of 40–60% after invasive candidiasis (Ghazi et al., 2019). Being the second or third most common cause of this disease (Tscherner et al., 2011; Fuller et al., 2019; Mari et al., 2019), *C. glabrata* successfully infects and prevails in the human host thanks to its ability to adapt, resisting antifungal treatment and the host stressful environment (Pais et al., 2019), often by being able to form biofilms (Cavalheiro and Teixeira, 2018). In order to develop antifungal resistance, *C. glabrata* resorts to the activation of different multidrug efflux pumps of the ATP-binding cassette (ABC) transporter superfamily and the major facilitator superfamily (MFS) (Costa et al., 2014a; Cannon and Holmes, 2015). Although CgCdr1 ABC transporter appears to play a primordial role in azole resistant clinical isolates, the upregulation of some of the MFS drug transporters has also been correlated with at least clotrimazole resistance in clinical isolates (Costa et al., 2016). The activation of several ABC transporters and MFS transporters is mostly due to the CgPdr1 transcription factor, regulator of multidrug resistance in *C. glabrata* (Costa et al., 2013b; Paul et al., 2014; Pais et al., 2016b; Whaley et al., 2018). This regulator may suffer gain-of-function (GOF) mutations that enhance the activation of such transporters (Moye-Rowley, 2019).

The ABC transporters have two transmembrane domains and two cytoplasmic nucleotide-binding domains, requiring energy from the hydrolysis of ATP, to cross substrates through the membrane. The ones with most dominant role in *C. glabrata* azole resistance are Cdr1, Cdr2, and Snq2 (Sanglard et al., 2009). While the role of ABC transporters has been well-characterized, only more recently MFS transporters have been studied with more detail. The MFS family is divided into two subgroups: Drug:H^+^ antiporter 1 (DHA1) and 2 (DHA2) transporter subfamilies, compromising transporters with 12 and 14 transmembrane segments, respectively; both with predicted transporters in the genome of pathogenic fungi: *Candida albicans, C. glabrata, Cryptococcus neoformans*, and *Aspergillus fumigatus* (Costa et al., 2014a). DHA transporters have important roles in *Saccharomyces cerevisiae* drug resistance (Sá-Correia et al., 2008; Santos et al., 2014) and, as more recently unraveled, in *C. glabrata* (Costa et al., 2014a). In the case of this pathogenic yeast, evidence for a role in antifungal resistance was so far obtained for the DHA transporters: CgAqr1, CgQdr2, CgFlr1_1 and CgFlr1_2, CgTpo1_1, CgTpo1_2, and CgTpo3 (Costa et al., 2013a,b, 2014b; Pais et al., 2016a, 2019). CgAqr1 has been shown to have a role in the resistance to fluconazole and clotrimazole, while being also important in the resistance to acetic acid, which interacts synergistically with these antifungals (Costa et al., 2013a). CgQdr2 transporter confers resistance to miconazole, tioconazole, clotrimazole, and ketoconazole, its expression depending directly on the Pdr1 transcription factor. In addition, CgQdr2 was shown to complement the role of quinidine resistance of its homolog in *Saccharomyces cerevisiae* (Costa et al., 2013b). CgTpo3 is involved in azole resistance but is also important for *C. glabrata* resistance to spermine, complementing its homolog in *S. cerevisiae* (Costa et al., 2014b). Under the control of CgPdr1, but also of CgYap1, transcription factors, are the genes encoding CgFlr1 and CgFlr2, shown to have a role in azole and 5-flucytosine resistance (Pais et al., 2016b). CgTpo1_1 and CgTpo1_2 also contribute to the development of azole resistance (Pais et al., 2016a).

Surprisingly, some of the DHA transporters were additionally found to play important roles in *C. glabrata* virulence. For example, CgTpo1_1 confers resistance to antimicrobial peptides, like histatin-5, thus making *C. glabrata* cells more virulent in a *Galleria mellonella* infection model (Santos et al., 2017). CgTpo1_2 is necessary for the survival of *C. glabrata* upon phagocytosis, and its expression is upregulated upon biofilm formation, while its deletion decreases the expression of adhesin-encoding genes during biofilm formation (Santos et al., 2017). CgDtr1 MFS transporter is not involved in drug resistance, but instead is necessary for *C. glabrata*'s full virulence in the infection model *G. mellonella*. CgDtr1 has a role in the survival upon phagocytosis, being necessary for the resistance to oxidative and acetic acid stress (Romão et al., 2017). More recently, CgQdr2 was also identified as playing a role in biofilm formation, although the underlying mechanisms remained elusive (Widiasih Widiyanto et al., 2019). All the roles described for MFS transporters highlight their promiscuity in transporting many different substrates, which appear to ultimately lead to unexpected roles in processes, such as virulence, immune system evasion, or biofilm formation.

In this work, we screened all the *C. glabrata* DHA1 MFS transporters for a possible role in biofilm formation. Previously characterized CgAqr1, CgQdr2, CgTpo1_1, CgTpo1_2, CgTpo3, CgFlr1_1, CgFlr1_2, and CgDrt1 transporters were studied, as well as two other MFS transporters, CgTpo4 (*CAGL0L10912g*) and CgYhk8 (*CAGL0J00363g*), which had not yet been characterized. The possible involvement of an ortholog of CaTec1 transcription factor in *C. glabrata*, CgTec1 (*CAGL0M01716g*), in the regulation of the MFS transporters during biofilm formation was also assessed. The deletion of those MFS transporters was evaluated in terms of the effect on the expression of given adhesins and on the changes in plasma membrane potential.

## Results

### Four, Out of the 10, Drug:H^+^ Antiporters in *C. glabrata* Are Required for Biofilm Development

**Figure 1:**
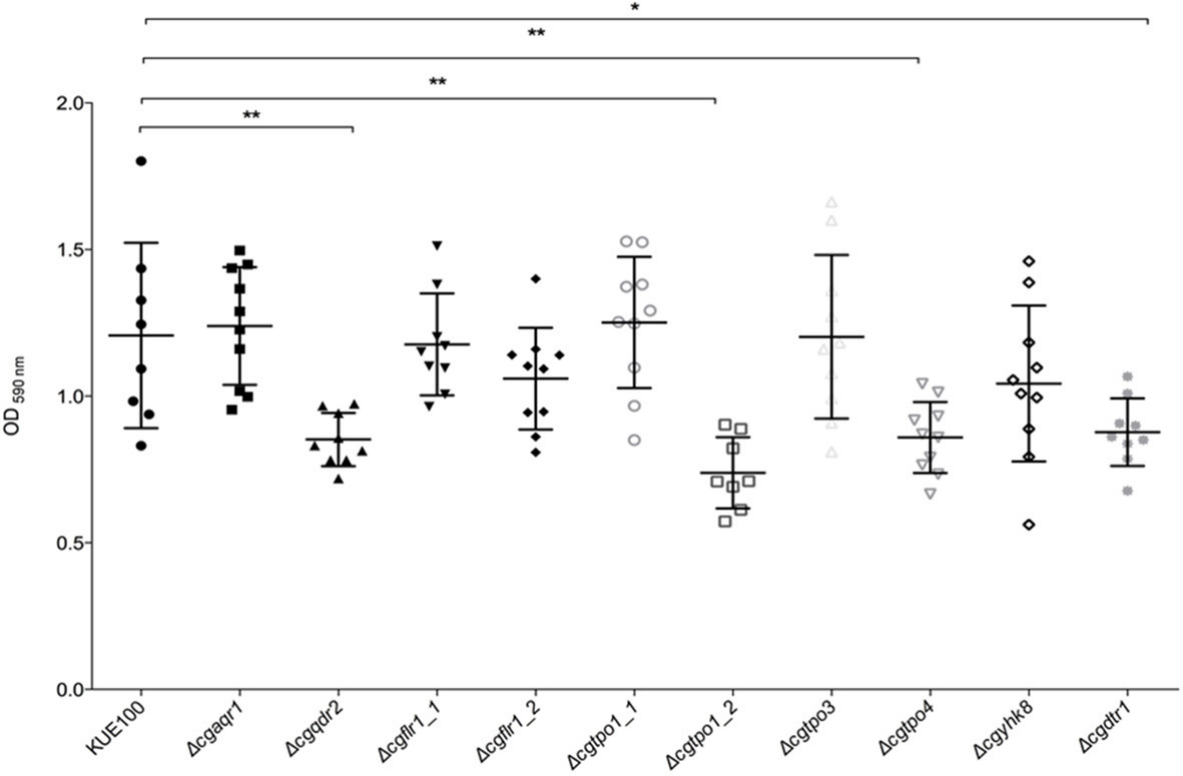
CgQdr2, CgTpo1_2, CgTpo4, and CgDtr1 are necessary for *C. glabrata* biofilm formation on polystyrene, in SDB pH 5.6. Assessment of 24 h biofilm formation was performed by crystal-violet assay in microtiter plates of *C. glabrata* KUE100, Δ*cgaqr1*, Δ*cgqdr2*, Δ*cgflr1_1*, Δ*cgflr1_2*, Δ*cgtpo1_1*, Δ*cgtpo1_2*, Δ*cgtpo3*, Δ*cgtpo4*, Δ*cgyhk8*, and Δ*cgdtr1* strains grown in SDB medium, pH 5.6. The data is displayed in a scatter dot plot, where each dot represents the level of biofilm formed in a sample. Horizontal lines indicate the average levels from at least three independent experiments. Error bars indicate standard deviations. *P < 0.05; **P < 0.01.

**Figure 2:**
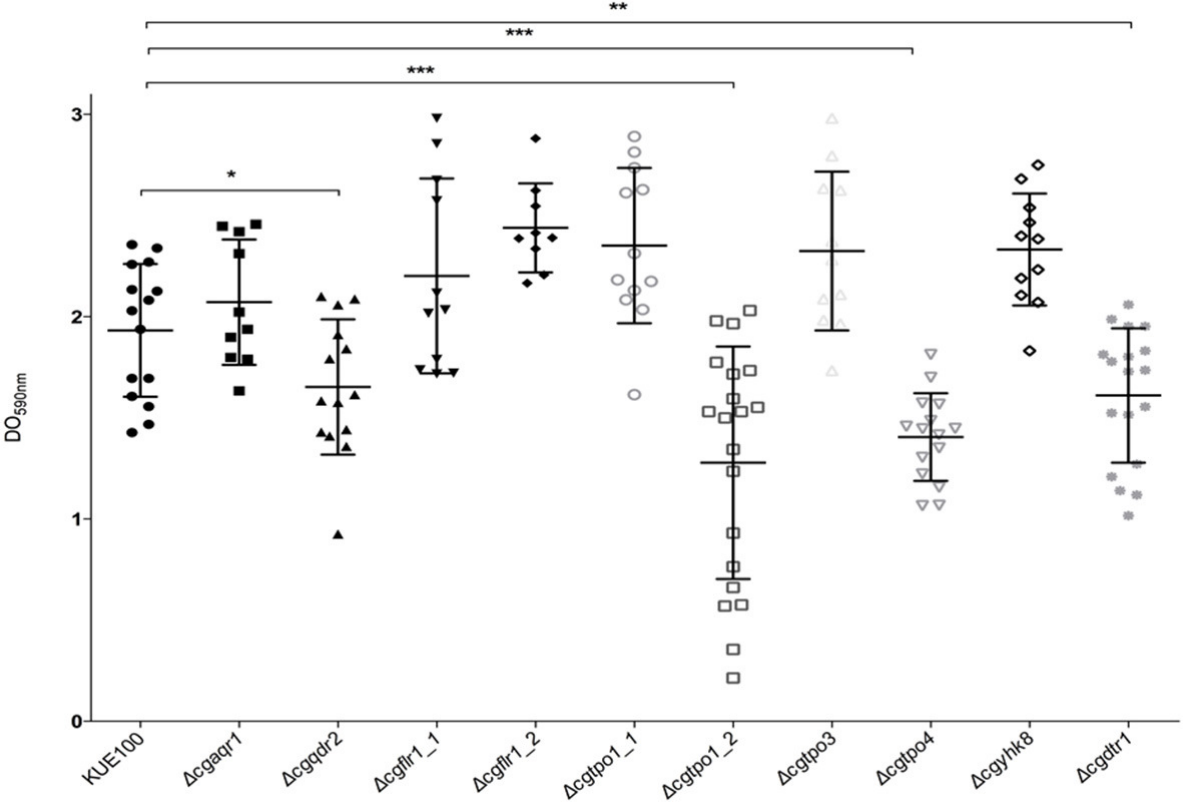
CgQdr2, CgTpo1_2, CgTpo4, and CgDtr1 are necessary for *C. glabrata* biofilm formation on polystyrene, in RPMI pH 4. Assessment of 24 h biofilm formation was performed by crystal-violet assay in microtiter plates of *C. glabrata* KUE100, Δ*cgaqr1*, Δ*cgqdr2*, Δ*cgflr1_1*, Δ*cgflr1_2*, Δ*cgtpo1_1*, Δ*cgtpo1_2*, Δ*cgtpo3*, Δ*cgtpo4*, Δ*cgyhk8*, and Δ*cgdtr1* strains grown in RPMI pH 4. The data is displayed in a scatter dot plot, where each dot represents the level of biofilm formed in a sample. Horizontal lines indicate the average levels from at least three independent experiments. Error bars indicate standard deviations. *P < 0.05; **P < 0.01; ***P < 0.001.

Given the previous implication of CgQdr2 and CgTpo1_2 in biofilm formation in *C. glabrata* (Santos et al., 2017; Widiasih Widiyanto et al., 2019), a systematic analysis of the possible involvement of all DHA1 transporters in this pathogenic yeast was carried out. The ability of the KUE100 wild-type strain and derived deletion mutants Δ*cgaqr1*, Δ*cgqdr2*, Δ*cgtpo1_1*, Δ*cgtpo1_2*, Δ*cgtpo3*, Δ*cgtpo4*, Δ*cgflr1_1*, Δ*cgflr1_2*, Δ*cgyhk8*, and Δ*cgdrt1* to form biofilms was assessed in SDB pH 5.6 and RPMI pH 4 media, on polystyrene, by the crystal-violet assay. Following previous studies (KucharíkovA et al., 2011; Gonçalves et al., 2016), RPMI medium was used at pH 4.0, given the acidic nature of some of the niches colonized by *Candida* species, as the vaginal tract (Owen and Katz, 1999; O'Hanlon et al., 2019). The deletion of *CgQDR2* and *CgTPO1_2* was confirmed to significantly decrease biofilm formation comparatively to the wild-type strain, in 30 and 40%, respectively, on both media, CgTpo1_2 playing a more prominent role (Figures 1, 2). Additionally, the deletion of *CgTPO4* and *CgDTR1* was also found to significantly decrease the ability to form biofilms on SDB pH 5.6 medium, in around 30% each, when compared to the wild-type strain (Figures 1, 2). The deletion of *CgFLR1_2, CgTPO1_1, CgTPO3*, and *CgYHK8* appears to lead to a slight increase in biofilm formation in RPMI medium, but this was not confirmed in SDB medium. Altogether, the obtained results expand current knowledge on the role for MFS transporters in biofilm formation, including two additional players in the process, CgTpo4 and CgDtr1.

### CgTec1 Transcription Factor Controls the Expression of *CgQDR2, CgTPO4*, and *CgDRT1* Genes in Early Biofilm Formation

**Figure 3:**
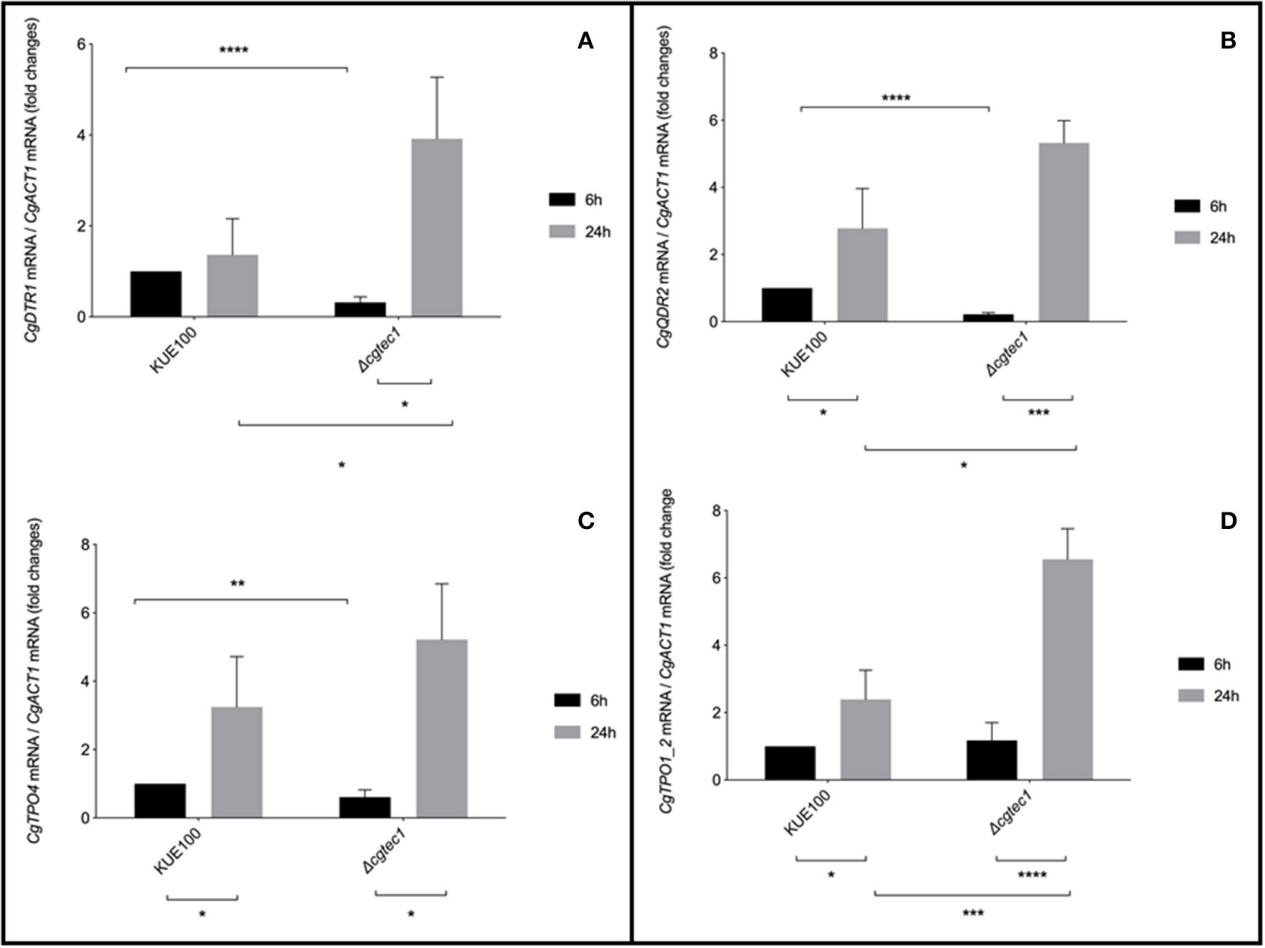
*CgQDR2, CgTPO4*, and *CgDTR1* genes are regulated by CgTec1 transcription factor, upon early biofilm formation. Shown are the transcript levels of **(A)** *CgDTR1*, **(B)** *CgQDR2*, **(C)** *CgTPO4*, and **(D)** *CgTpo1_2* in the *C. glabrata* wild-type strain KUE100 and in the derived deletion mutant Δ*cgtec1*, in 6 and 24 h of biofilm formation conditions on polystyrene surface in liquid SDB medium, pH 5.6. Transcript levels were assessed by quantitative RT-PCR, as described in Materials and Methods. Values are averages of results from at least three independent experiments. Error bars represent standard deviations. *P < 0.05; **P < 0.01, ***P < 0.001; ****P < 0.0001.

Although in *C. glabrata* very little is known about the regulation of biofilm formation, in *C. albicans* one of the major regulators of biofilm formation is CaTec1 transcription factor (Schweizer et al., 2000; Nobile et al., 2012; Daniels et al., 2015; Panariello et al., 2017). The deletion mutant of the predicted ortholog of CaTec1 in *C. glabrata*, encoded by ORF *CAGL0M01716g* and here named CgTec1, was used to assess its possible role controlling the expression of these MFS transporters during early (6 h) and mature (24 h) stages of biofilm formation. Although for the majority of *Candida* spp, 48 h are required to reach mature biofilms, *C. glabrata* biofilms are apparently at an intermediate maturation phase at 24 h of *in vitro* biofilm formation, where a confluent monolayer is already obvious, with the presence of extracellular matrix (Kucharikova et al., 2014). Upon 24 h of biofilm formation, the expression of *CgQDR2, CgTPO1_2*, and *CgTPO4* genes is upregulated in the KUE100 wild-type strain, comparatively to 6 h of biofilm formation (Figure 3). Moreover, the deletion of *CgTEC1* gene, leads to a severe decrease in the expression of *CgQDR2, CgTPO4*, and *CgDRT1* at 6 h of biofilm formation, but not at 24 h (Figure 3). These results indicate that CgTec1 is required for the activation of *CgQDR2, CgTPO4*, and *CgDTR1* transcription in the early stages of biofilm formation. This suggests a specific window period in which these transporters act for the benefit of biofilm formation, the early stage of biofilm, under the control of the CgTec1 transcription factor.

### The Transcript Levels of Adhesin Encoding Genes Are Repressed in Δ*cgqdr2*, Δ*cgtpo1_2*, Δ*cgtpo4*, and Δ*cgdrt1* Biofilms

**Figure 4:**
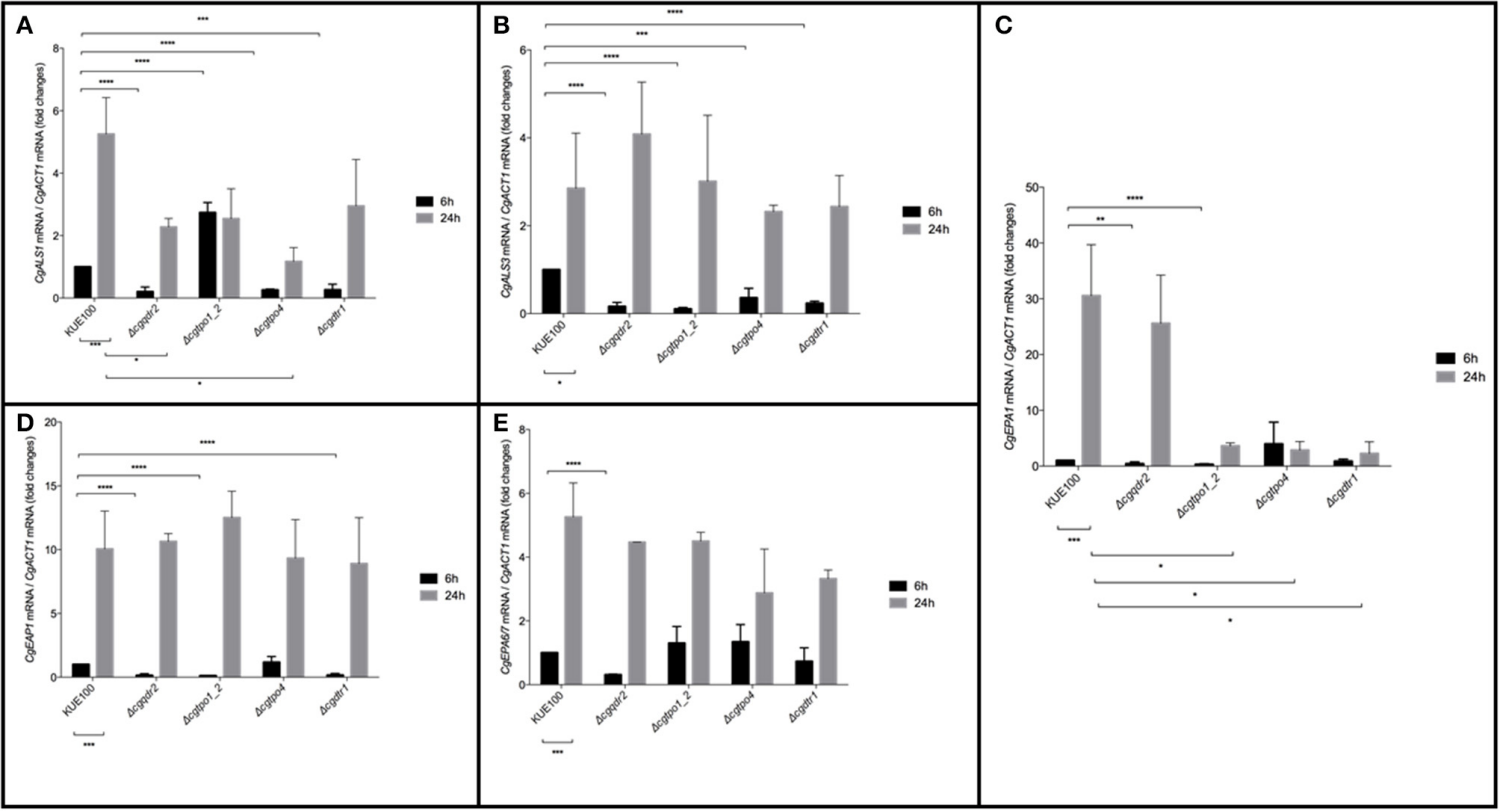
Effect of *CgQDR2, CgTPO1_2, CgTPO4*, and *CgDTR1* gene deletion in the expression of adhesin encoding genes *CgALS1, CgEAP1, CgEPA1*, and *CgEPA6*/7 during biofilm formation. Comparison of the variation of the *CgALS1* **(A)**, *CgALS3* **(B)**, *CgEPA1* **(C)**, *CgEAP1* **(D)**, and *CgEPA6*/*7* **(E)** transcript levels in KUE100 *C. glabrata* wild-type cells and Δ*cgqdr2*, Δ*cgtpo1_2*, Δ*cgtpo4*, and Δ*cgdrt1* mutant cells, after 6 h (black bars) or 24 h (gray bars) of biofilm development. The presented transcript levels were obtained by quantitative RT-PCR and are normalized to the *CgACT1* mRNA levels, relative to the values registered in wild-type cells after 6 h of biofilm development (6 h). The indicated values are averages of at least three independent experiments. Error bars represent the corresponding standard deviations. *P < 0.05; **P < 0.01; ***P < 0.001; ****P < 0.0001.

Considering the importance of these MFS transporters on biofilm formation, their impact in the expression of a set of 5 adhesin encoding genes, *CgALS1, CgEAP1, CgEPA1, CgEPA6*, and *CgEPA7*, linked to adherence and biofilm formation (de Groot et al., 2013), was assessed. Gene expression was measured at 6 and 24 h of biofilm development in the KUE100 wild-type strain and in the Δ*cgqdr2*, Δ*cgtpo1_2*, Δ*cgtpo4*, and Δ*cgdrt1* deletion mutants (Figure 4). The relative expression of the genes in the wild-type strain KUE100 at 6 h of biofilm growth were used as a reference. The results regarding the expression of *CgEPA6* and *CgEPA7* are presented combined given that they share 92% homology and their transcript levels are indistinguishable.

The expression of all selected adhesin encoding genes is upregulated upon 24 h of wild-type strain biofilm formation comparatively to 6 h of biofilm formation. As described previously (Santos et al., 2017), upon the deletion of *CgTPO1_2*, the transcript levels of *CgALS1, CgEAP1*, and *CgEPA1* are decrease comparatively to the wild-type strain, in at least one of the time points (Figures 4A,C,D). In turn, deletion of *CgQDR2* gene leads to a repression of the expression of all adhesin-encoding genes at 6 h of biofilm formation (Figures 4A–E). *CgTPO4* deletion leads to the repression of *CgALS1, CgALS3*, and *CgEPA1* genes, at the same time point (Figures 4A,B), while the deletion of *CgDTR1* results in a decrease of expression of the *CgALS1, CgALS3, CgEAP1*, and *CgEPA1* (Figures 4A,B,D).

Such influence on the expression of different adhesin-encoding genes, especially upon 6 h of biofilm formation, indicates once again that CgQdr2, CgTpo1_2, CgTpo4, and CgDrt1 have an important role in the early stage of this process in *C. glabrata* and that they appear to act mostly by indirectly delaying adhesin gene up-regulation.

### Membrane Potential Is Increased in the Absence of CgQdr2, CgTpo4, and CgDtr1

**Figure 5:**
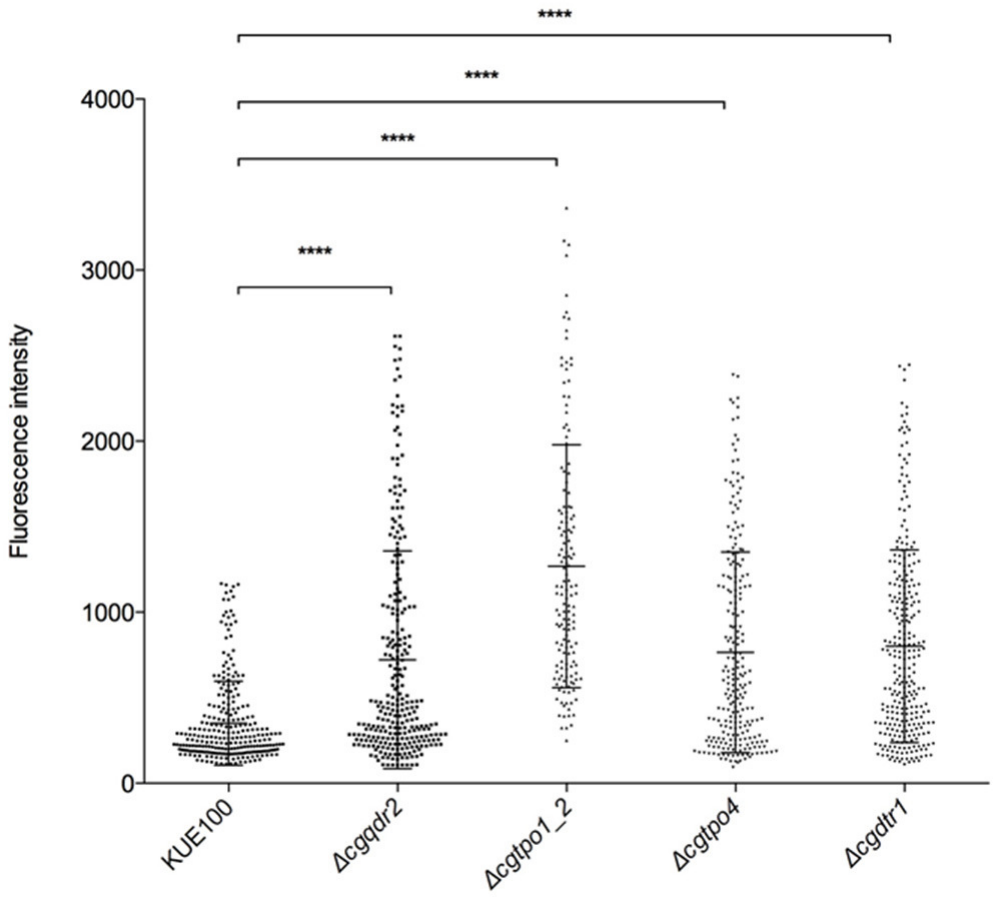
The deletion of *CgQDR2, CgTPO1*_2*, CgTPO4*, and *CgDTR1* increases plasma membrane potential. *C. glabrata* wild-type KUE100 and derived Δ*cgqdr2*, Δ*cgtpo1_2*, Δ*cgtpo4*, and Δ*cgdrt1* mutant cells were grown to mid-exponential phase of growth in YEPD medium. These cells were incubated with the fluorescent dye DiOC6(3), whose uptake and accumulation depends on the plasma membrane potential, and fluorescence microscopy was used to measure the fluorescence intensity of individual cells. A scatter dot plot representation of the data is shown, where each dot represents the fluorescence intensity of each individual cell. The average level of fluorescence intensity, considering at least three independent experiments, and at least 100 cells per experiment, is indicated by the black line (–), standard deviation being represented by the error bars. ****P < 0.0001.

Given the clear influence of CgQdr2, CgTpo1_2, CgTpo4, and CgDtr1 in early biofilm formation, we further investigated how these transporters might be contributing for the initiation of this process. It has been described that the environment is a key factor that modulates the adherence and biofilm formation, especially in terms of the deficiency of certain nutrients (Verstrepen and Klis, 2006; Fisher et al., 2011; Riera et al., 2012). The plasma membrane potential directly affects the secondary transporters responsible for nutrient uptake, who have the membrane potential as a driving force. Therefore, changes in membrane potential are likely to affect cell proficiency in the uptake of given nutrients (Goossens et al., 2000), thus influencing the signaling leading to biofilm formation. Having this in mind, we assessed the effect of the absence of each of the genes in study in *C. glabrata* membrane potential. The plasma membrane potential of KUE100 wild-type strain and Δ*cgqdr2*, Δ*cgtpo1_2*, Δ*cgtpo4*, and Δ*cgdrt1* deletion mutant cells was monitored through the accumulation of the fluorescent dye DiOC6(3) (Cabrito et al., 2011). All deletion mutants were found to exhibit increased membrane potential comparatively to the wild-type strain (Figure 5), an effect already described for CgTpo1_2 (Santos et al., 2017). These results suggest that CgQdr2, CgTpo1_2, CgTpo4, and CgDtr1 are important for plasma membrane potential homeostasis, which is likely to affect the cellular perception of nutrient availability, a key step in the triggering of biofilm formation.

## Discussion

In this study, the previously characterized multidrug transporters CgAqr1, CgQdr2, CgTpo1_1, CgTpo1_2, CgTpo3, CgFlr1_1, CgFlr1_2, and CgDrt1 transporters, as well as two others, CgTpo4 and CgYhk8, all belonging to the DHA1 family, were screened for a possible role in biofilm formation. This systematic screening was driven by the observation that the majority of the MFS transporters characterized so far appear to transport additional substrates beyond drugs (Costa et al., 2013a,b, 2014b; Pais et al., 2016a, 2019), which affect *C. glabrata* pathogenesis and virulence (Romão et al., 2017; Santos et al., 2017).

Besides confirming the previously identified role of CgQdr2 (Widiasih Widiyanto et al., 2019) and CgTpo1_2 (Santos et al., 2017) in biofilm formation, two new DHA transporters were linked to this process: CgTpo4 and CgDtr1. This apparently widespread role of DHA transporters in biofilm formation is consistent with the previously described implication of the DHA transporters CaQdr1, CaQdr2, and CaQdr3 in biofilm formation in *C. albicans*. Deleting all *QDR* genes in *C. albicans* leads to clear defects in the architecture and thickness of the biofilm, which is suggested to be related to the remodeling of lipids *C. albicans* cells suffer upon the loss of such genes (Shah et al., 2014). The role of CgTpo1_2 in biofilm formation was also linked with its effect in ergosterol and fatty acid content, but mostly through its influencing over plasma membrane potential (Santos et al., 2017). Interestingly, CgQdr2, CgTpo4, and CgDtr1 were also found in this study to affect the membrane potential. The alteration of plasma membrane potential by DHA transporters may be related to their role in the transport of small charged molecules, including cations and polycations (Vargas et al., 2007). In *Saccharomyces cerevisiae*, MFS transporters are known to be involved in this type of transport. For instance, ScQdr2 is involved in K^+^ import (Vargas et al., 2007) and ScTpo1-4 (Tomitori et al., 2001) and ScQdr3 (Teixeira et al., 2011) are implicated in the export of polyamines. Given that increased plasma membrane potential is implicated in higher secondary transport activity (Eddy and Hopkins, 1998), nutrient uptake capacity may be modified upon the absence of each transporter. This may possibly affect biofilm formation as has been described for bacterial MFS transporters. Indeed, bacterial biofilms are influenced by the nutrients in the environment, given that the uptake of given nutrients acts as a positive or negative signal for the initiation of this process. Therefore, a key role in biofilm formation was identified for bacterial MFS transporters, responsible for nutrient uptake (Pasqua et al., 2019). It is, thus, likely that the same phenomenon may also occur in yeast biofilms.

Given that these transporters have a clear effect on the plasma membrane, we hypothesized if they might influence the presence of given proteins on the plasma membrane and cell wall, involved on biofilm formation. With this in mind, we assessed the expression of adhesin-encoding genes upon the deletion of *CgQDR2, CgTPO1_2, CgTPO4*, and *CgDTR1* genes, in biofilm conditions. *CgALS1* and *CgEPA1* expression was found to be decrease upon the absence of all transporters, for at least one of the time points tested (6 and 24 h). In addition, more adhesins were found to be repressed upon the specific absence of each transporter, highlighting the clear influence MFS transporters have on the presence of adhesins in the cell envelop. It is possible that the absence of these MFS transporters alters the perception of nutrient availability, delaying the activation of adhesin-encoding genes, and ultimately leading to defects on the capacity of *C. glabrata* to adhere and form biofilms.

Interestingly, in *C. albicans*, MFS transporters, CaMdr1 and CaQdr1, have also been linked to biofilm formation and cell dispersion, being up-regulated in both conditions. It is suggested in the work of Uppuluri et al. (2018) that the upregulation of these and other types of transporters is related to the reprogramming of dispersal cells to acquire nutrients and be able to attach and survive in nutrient-starved niches of the host (Uppuluri et al., 2018). It would be interesting to test if CgQdr2, CgTpo1_2, CgTpo4, and CgDtr1 have a role on this last phase of biofilm formation. Nevertheless, our results suggest that their activation is more significant in early stages of biofilm formation than the later.

Although the specific role of these transporters may not yet be clear, *CgQDR2, CgTPO4*, and *CgDTR1* genes were found to be activated by the CgTec1 transcription factor in early stages of biofilm formation. CgTec1 has not yet been characterized in *C. glabrata* but it seems to be involved on the regulation of biofilm in this yeast, like its ortholog's role in the regulation of the same process in *C. albicans*. CaTec1 has a minor role in adhesion but is required for the formation of the several layers of cells and hyphal formation, and influences the thickness and integrity of the biofilm (Schweizer et al., 2000; Daniels et al., 2015). CaTec1 is also necessary for the full virulence of *C. albicans* (Yano et al., 2016). It is possible that the CgTec1 transcription factor in *C. glabrata* may have important roles as is ortholog, starting by the control of these transporters at the beginning of biofilm formation.

Based on these results our current model is that the deletion of *CgQDR2, CgTPO1_2, CgTPO4*, and *CgDTR1* genes leads to an increase in plasma membrane potential, which possible affects nutrient uptake, influencing the signaling that triggers cellular adhesion, eventually compromising *C. glabrata* biofilm formation. Moreover, CgQdr2, CgTpo4, and CgDtr1 expression appears to be controlled by one of the predicted regulators of biofilm formation, CgTec1, highlighting their role in the process. Altogether, DHA transporters appear to be in the crossroad between drug resistance, biofilm formation as well as additional pathogenesis traits (Cavalheiro et al., 2018), highlighting their potential impact in the success of *C. glabrata* infections and in the design of novel antifungal therapeutic approaches.

## Materials and Methods

### Strains, Plasmids, and Growth Medium

*Candida glabrata* KUE100 (Ueno et al., 2007) strain was used in this study. The *Candida glabrata* Δ*cgtpo1_1*, Δ*cgtpo1_2*, Δ*cgaqr1*, Δ*cgqdr2*, Δ*cgflr1_1*, Δ*flr1_2*, Δ*cgtpo3*, and Δ*cgdrt1* deletion mutants, constructed in previous studies (Costa et al., 2013a,b, 2014b; Pais et al., 2016a; Romão et al., 2017), were also used. Δ*cgtec1*, Δ*cgtpo4*, and Δ*cgyhk8* deletion mutants were constructed as described in the next section.

*Candida glabrata* cells were cultivated in rich YEPD medium, containing per liter: 20 g D-(+)- glucose (Merk, Darmstadt), 20 g bacterial-peptone (LioChem, Conyers, Georgia) and 10 g of yeast-extract (Difco, Detroit, Michigan). Sabouraud's Dextrose Broth (SDB) pH 5.6, used for *C. glabrata* planktonic and biofilm cultivation, contains 40 g glucose (Merk, Darmstadt) and 10 g peptone (LioChem, Conyers, Georgia) per liter. RPMI 1640 medium pH 4, used for *C. glabrata* planktonic and biofilm cultivation, contains 10,4 g RPMI 1640 (Sigma, Darmstadt), 34,5 g MOPS (Sigma, Darmstadt) and 18 g glucose (Merck, Darmstadt) per liter.

### Disruption of the *C. glabrata CgTPO4, CgYHK8*, and *CgTEC1* Genes (ORF *CAGL0L10912g CAGL0J00363g* and *CAGL0M01716g*)

The deletion of the *CgTPO4, CgYHK8*, and *CgTEC1* genes was carried out in the parental strain KUE100, using the method described by Ueno et al. (2007). The target genes were replaced by a DNA cassette including the *CgHIS3* gene, through homologous recombination. The DNA cassette was amplified with PCR for which gene disruption primers (Table 1) including homologous sequences at 5′ end and as a template the pHIS906 plasmid including CgHIS3 were used. Transformation was performed with the DNA cassette as described previously (Ueno et al., 2007). Recombination locus and gene deletion were verified by PCR using the primers indicated in Table 1.

**Table 1:** List of primers used in this study.

**Name**	**Sequence (5′-3′)**
***CgTPO4***, ***CgYHK8*** **gene disruption**	
Δ*CgTPO4_Fw*	GAACTGGTGAAATATAGTATAAGCGTTACAAAGCGAATAACGAATACATACACCACGGCCGCTGATCACG
Δ*CgTPO4_Rv*	AAGAGCAAAAGTATTCAATTTTTTAAAAATTTAAAGCAAATCGAAAAAAAGGACTACATCGTGAGGCTGG
Δ*CgYHK8_Fw*	TTGCTCGACTTCTATATCTTACACTATTACACAACCAAAATCAGCAACAATAGAAAGGCCGCTGATCACG
Δ*CgYHK8_Rv*	CTAAAAAAAGATCAAATGGTTCGTGCTGCTGTTATATTCAGGGATAAGGCAGATTACATCGTGAGGCTGG
Δ*CgTEC1_Fw*	AAGAGTACTAATACACATCGTACTCCCCCCCACAAATAACGCCCTCAATCTATATTGGCCGCTGATCACG
Δ*CgTEC1_Rv*	TCAGCAAAACATTTCTGCAGAAAAAATAAAAATGTAGCATTCCTACATCTCTCTCACATCGTGAGGCTGG
**Gene disruption confirmation**	
Δ*CgTPO4_Fw_conf*	CAAGTTGGTGATACTAATAGCA
Δ*CgTPO4_Rv_conf*	CACTTCACTCAAGGGAGC
Δ*CgYHK8_Fw_conf*	GATGAAGGACTCAGATTCG
Δ*CgYHK8_Rv_conf*	CCAGGTTGTCAGGCATTG
Δ*CgTEC1_Fw_conf*	GACAGCTCGGTATCAGATAGGT
Δ*CgTEC1_Rv_conf*	GTGGAGATGATGCTTTCGAAGA
**RT-PCR experiments**	
*CgACT1_Fw*	AGAGCCGTCTTCCCTTCCAT
*CgACT1_Rv*	TTGACCCATACCGACCATGA
*CgALS1Fw*	GAG CTC AAT GCA GAA GTG TAC TTT G
*CgALS1_Rv*	GAT CTG ATT GTG GTA TAA AAG TGG TCA T
*CgALS3_Fw*	GTTGACCCATTTCGTGGAAAA
*CgALS3_Rv*	GAAGGCCATAATTTCACAGTCAGA
*CgEPA1_Fw*	TTG ATT GCT GCA GAA GGG ATT
*CgEPA1_Rv*	ATG GCG TAG GCT TGA TAA TTT CC
*CgEAP1_Fw*	CAA CAC CAG CCC AAT CAA ATG
*CgEAP1_Rv*	CGG AAG ACA TCG TTA ATG AAG GA
*CgEPA6/7_Fw*	TTC CCT TCG CAA CTT ACA CAA CT
*CgEPA6/7_Rv*	GAA GCA CTC CCA CTG CTA GAG TAA
*CgTPO1_2_Fw*	AGGACCCGCTCTATCGAAAAA
*CgTPO1_2_Rv*	GCTGCGACTGCTGACTCAAC
*CgTPO4_Fw*	TCGTTGGCCCATTTTTGG
*CgTPO4_Rv*	GCAAACCCGCGATGA
*CgDTR1_Fw*	GGAGCCAAAATGAGAATGATATGTC
*CgDTR1_Rv*	ACCACCTTGAAATCGGTGATG
*CgQDR2_Fw*	TCACTGCATAGTTTCATATCGGACTA
*CgQDR2_Rv*	CAACTTCAGATAGATCAGGACCATCA

### Biofilm Quantification

*Candida glabrata* strains were tested for their capacity for biofilm formation, recurring to the crystal-violet method (Pathak et al., 2012). For that, the *Candida glabrata* strains were grown in SDB medium and harvested by centrifugation at mid-exponential phase. The cells were inoculated with an initial OD_600nm_ = 0,05 ± 0,005—corresponding to 5 × 10^5^ CFU/ml—in 96-well polystyrene microtiter plates (Greiner) in either SDB (pH 5.6) or RPMI (pH 4) media. Cells were cultivated at 30°C during 15 ± 0,5 h with mild orbital shaking (70 rpm), as before (Melo et al., 2006; Pathak et al., 2012; Santos et al., 2017; Cavalheiro et al., 2019). After the incubation time, each well was washed three times with 200 μL of deionized water to remove cells not attached to the biofilm matrix. Then, 200 μL of a 1% crystal-violet (Merck, Darmstadt) alcoholic solution was used to stain the biofilm present in each well. Following 15 min of incubation with the dye, each well was washed with 250 μL of deionized water. The stained biofilm was eluted in 200 μL 96% (v/v) ethanol and the absorbance of each well was read in a microplate reader at the wavelength of 590 nm (SPECTROstar Nano, BMG Labtech, Ortenberg).

### Gene Expression Measurement

The transcript levels of *CgTPO1_2, CgTPO4, CgQDR2*, and *CgDTR1*, and of the adhesin encoding genes *CgALS1, CgEAP1, CgEPA1, CgEPA6*, and *CgEPA7* were determined by quantitative real-time PCR (RT-PCR). Total RNA was extracted from cells grown in biofilm. 40 mL of fresh RPMI 1640 (pH 4) was placed in square polystyrene petri plates (Greiner), and cells were added so that the initial OD_600nm_ = 0.05 ± 0,005. The plates were incubated at 30°C and 30 rpm during 6 and 24 h to analyse both young and mature biofilm development for each strain under analysis. A lower agitation speed was used in this case to prevent spilling of part of the culture. It does not compromise aeration, as the surface area of the used petri dishes is much higher than that in microtiter plates. At the end of each period the supernatant was discarded, and the biofilm was removed with a metal spatula. Samples were centrifuged to remove excess water and frozen at −80°C until RNA extraction. Planktonic growing cells, used as control, were cultivated in RPMI 1640 (pH 4) with orbital shaking (250 rpm) at 30°C and harvested by centrifugation at comparable times.

For total RNA extraction, the hot phenol method was applied (Köhrer and Domdey, 1991). Total RNA was converted to cDNA for the real-time Reverse-Transcription PCR (RT-PCR) using the MultiScribe Reverse Transcriptase kit (Applied Biosystems, Foster City, California) and the 7500 RT-PCR thermal cycler block (Applied Biosystems, Foster City, California). The quantity of cDNA for subsequent reactions was kept at ca. 10 ng. The real time PCR step was carried out using adequate primers (Table 1) designed by the Primer Express™ Software v3.0.1, SYBR Green^®^ reagents (Applied Biosystems, Foster City, California) and the 7500 RT-PCR thermocycler block (Applied Biosystems, Foster City, California). Default parameters set by the manufacturer were followed, and fluorescence was detected by the instrument and plotted in an amplification graph (7500 Systems SDS Software, Applied Biosystems, Foster City, California). *CgACT1* gene transcript level was used as an internal reference.

### Estimation of Plasma Membrane Potential

The estimation of the plasma membrane potential was carried out by measuring the fluorescence intensity of cells exposed to the fluorescent carbocyanine 3,3′-Dihexyloxacarbocyanine Iodide [DiOC6(3)] (Cabrito et al., 2011). Cells were cultivated in SDB media until mid-exponential phase, washed twice in Mes/glucose buffer [10 mM Mes, 0.1 mM MgCl_2_ and 20 g/l glucose (pH 5,6)] and resuspended in Mes/glucose buffer, supplemented with 250 nM DiOC6(3) (Molecular Probes, Eugene, Oregon), followed by incubation in the dark for 30 min at 30°C with orbital agitation (250 rpm). After centrifugation cells were observed with a Zeiss Axioplan microscope equipped with adequate epifluorescence filters (BP450-490 and LP520). Fluorescence emission was collected with a CCD (charge-coupled device) camera (CoolSNAPFX, Roper Scientific Photometrics, Tucson, Arizona). For the excitation of the fluorescent molecule, radiation with a wavelength of 480 nm was used. The images were analyzed using MetaMorph 3.5. The fluorescence intensity values, obtained pixel-by-pixel in the region of interest, were calculated for a minimum of 100 cells per experiment, considering a minimum of 3 independent experiments, per strain.

### Statistical Analysis

Statistical analysis was performed using Graphpad Prism Software version 6.0 and analyzed with Student's *t*-test. *P*-values equal or inferior to 0.05 were considered statistically significant.

## Data Availability Statement

All datasets generated for this study are included in the article/supplementary material.

## Author Contributions

RS, MC, and CC conducted most of the experiments. AT-N and MO played a key role in strain design and construction. MC, RS, and MT wrote the paper. HC and MT conceived and supervised all the work.

### Conflict of Interest

The authors declare that the research was conducted in the absence of any commercial or financial relationships that could be construed as a potential conflict of interest.
